# Is It Possible?

**Published:** 1920-10-23

**Authors:** 


### A STATE NURSING SERVICE.

### Is it Possible?

It is scarcely an accident that those who sit in high places in the hospital world decline altogether to discuss the pay and prospects of the nurse, or else, in so doing, sedulously avoid definiteness or detail. The probable reason for this attitude is that it is the only one they can afford. A man does not regale his family with guide-books to the Riviera if he knows that a week-end at Margate is his *ne plus ultra*. Every nurse may not have noticed it, but I think it will be found on examination that while prominent men and women, and prominent newspaper editors also, usually refer in tones of optimism to the nurse's outlook, none of them mentions figures or dates. The nearest approach was the salary scale of the College of Nursing. I am afraid that Hospital Boards found too many immediate and urgent claims on their attention to permit of their giving this useful and interesting document the attention which it deserved. At the moment it is probably pigeon-holed and a little dusty! A high authority declares that owing to the unavoidable increase in expenditure, and the falling-off of subscriptions, the financial position of voluntary hospitals has become "alarming." The medical and allied professions are arranging a conference to be held early in November to discuss the situation, and, if possible, to save the hospitals from being thrown on the rates.

I hope it is an accidental omission, and one which will be rectified, that there is no mention of any Society of Nurses summoned to the conference at which are to be represented three Royal Colleges of Physicians and Surgeons, the Society of Apothecaries, and the British Medical Association. It is extremely doubtful, however, even if represented, that the subject of the welfare of nurses will be seriously discussed, or, indeed, mentioned at all at this conference. The doctors, with the best possible of wishes to nurses, cannot afford to put this on the agenda.

### Semper Idem.

It is always the same. You must not mention an eight-hours' day because of the housing difficulty. Put it off to a more convenient season. An eight-hours' day, a promising career, a pension for the disabled—all this will come, but I am afraid, according to the portents, it will be in the Never Never Land. When hospitals were fairly well-to-do the nurse's cupboard was, generally speaking, pretty bare. What is to happen when they are bordering on bankruptcy? With the strongest desire to maintain an optimistic spirit, I confess that I see no grounds for hope of anything better than a hand-to-mouth existence. This month's salary may be paid. What of next month's? The profession is not on the upward way, and probationers are not likely to be dazzled.

Is it not better to face the music? The nurse has been provided with one great entertainment in illusions. The time has come, I respectfully submit, to provide her with something tangible, or else to tell her the worst. My firm conviction, after much reflection, is that the present system offers her no outlook. The economic condition of British nursing, like the financial state of the hospitals, is indeed "alarming."

### No Jeremiad.

No! This is not a jeremiad. It is merely a frankly expressed opinion that a system which has been in vogue for half a century, which never was a success, shows unmistakable signs of being unable to survive post-war conditions. I do not refer to the voluntary hospital, but to the maintenance of an effectual national nursing service out of the funds of voluntary hospitals. An effectual nursing service cannot be maintained unless there is provided for the members the possibility of a successful career, and a satisfactory provision for infirmity or disablement. Preaching sermons to the rising generation is vain. The demand on them to work for a lower reward than other workers receive is obviously unfair, and realising this they simply will not respond to the call. Why should they?

The suggestion that I have to offer is that there is a way out *via* the Ministry of Health. In this Department of State is vested the responsibility of the nation's health. Why should not the Health Minister be called upon to organise a national nursing service on lines corresponding with the Army Nursing Service?

The question is a very large one, and cannot be dismissed with a simple "Yes" or "No." I can see quite a number of difficulties in the way. But before facing these I should like to see the question discussed as to whether the country expects the Ministry of Health to ensure the maintenance of an efficient body of trained nurses and midwives. Physicians and surgeons are interested in the voluntary hospital. The nurse is far more intimately interested. It is her world, and in the past it has made a heroic and a successful effort to keep the standard high. There is, of course, no limit to what may be achieved by enlightened rating authorities, but in hospital enterprise it will be hard to get away from the workhouse trail.

We have, I think, silently arrived at a crisis, and the question that has to be settled is a national one. I should very much like to see it discussed by responsible nursing authorities, whether in their opinion the principle is sound that the Minister of Health should be called upon to provide a State nursing service.

If this is conceded all difficulties resolve themselves into matters of detail. IERNE.

[The question thus raised is indeed a large one, as "Ierne" candidly admits. We doubt whether the scheme is practical politics for the moment, and certainly it would arouse opposition in many quarters. While "Ierne's" strictures on the treatment of nurses by hospital authorities can undoubtedly be justified in several institutions, it is hardly a correct generalisation to assume that British hospitals as a class are unmindful of their obligations to the devoted women who serve them.—Editor The Hospital.]

